# An epigenetic clock for gestational age at birth based on blood methylation data

**DOI:** 10.1186/s13059-016-1068-z

**Published:** 2016-10-07

**Authors:** Anna K. Knight, Jeffrey M. Craig, Christiane Theda, Marie Bækvad-Hansen, Jonas Bybjerg-Grauholm, Christine S. Hansen, Mads V. Hollegaard, David M. Hougaard, Preben B. Mortensen, Shantel M. Weinsheimer, Thomas M. Werge, Patricia A. Brennan, Joseph F. Cubells, D. Jeffrey Newport, Zachary N. Stowe, Jeanie L. Y. Cheong, Philippa Dalach, Lex W. Doyle, Yuk J. Loke, Andrea A. Baccarelli, Allan C. Just, Robert O. Wright, Mara M. Téllez-Rojo, Katherine Svensson, Letizia Trevisi, Elizabeth M. Kennedy, Elisabeth B. Binder, Stella Iurato, Darina Czamara, Katri Räikkönen, Jari M. T. Lahti, Anu-Katriina Pesonen, Eero Kajantie, Pia M. Villa, Hannele Laivuori, Esa Hämäläinen, Hea Jin Park, Lynn B. Bailey, Sasha E. Parets, Varun Kilaru, Ramkumar Menon, Steve Horvath, Nicole R. Bush, Kaja Z. LeWinn, Frances A. Tylavsky, Karen N. Conneely, Alicia K. Smith

**Affiliations:** 1 Genetics and Molecular Biology Program, Emory University, Atlanta, GA USA; 2 Murdoch Childrens Research Institute and Department of Paediatrics, University of Melbourne, Parkville, Victoria 3052 Australia; 3 The Royal Women’s Hospital, Murdoch Childrens Research Institute and University of Melbourne, Parkville, Victoria 3052 Australia; 4 Section of Neonatal Genetics, Danish Centre for Neonatal Screening, Department for Congenital Disorders, Statens Serum Institut, Artillerivej 5, DK-2300 Copenhagen S, Denmark; 5 The Danish Neonatal Screening Biobank, Department for Congenital Disorders, Statens Serum Institut, Artillerivej 5, DK-2300 Copenhagen S, Denmark; 6 National Centre for Register-based Research, School of Business and Social Sciences, Aarhus University, Fuglesangs Allé 4, 8210 Aarhus V, Denmark; 7 Institute of Biological Psychiatry, Sct. Hans Mental Health Center, Copenhagen Mental Health Services, iPSYCH - The Lundbeck Foundation’s Initiative for Integrative Psychiatric Research, Boserupvej, DK-4000 Roskilde, Denmark; 8 Department of Psychology, Emory University, Atlanta, GA USA; 9 Department of Human Genetics, Emory University School of Medicine, Atlanta, GA USA; 10 Department of Psychiatry & Behavioral Sciences, Emory University School of Medicine, Atlanta, GA USA; 11 Departments of Psychiatry & Behavioral Sciences and Obstetrics & Gynecology, University of Miami Miller School of Medicine, Miami, FL USA; 12 Departments of Psychiatry & Behavioral Sciences, Pediatrics, and Obstetrics & Gynecology, University of Arkansas for Medical Sciences, Little Rock, AR USA; 13 Laboratory of Environmental Precision Biosciences, Columbia University Mailman School of Public Health, New York, NY USA; 14 Department of Preventive Medicine, Icahn School of Medicine at Mount Sinai, New York, NY USA; 15 Center for Nutrition and Health Research, National Institute of Public Health, Cuernavaca, Morelos Mexico; 16 Department of Environmental Health, Harvard T.H. Chan School of Public Health, Boston, MA USA; 17 Department of Translational Research in Psychiatry, Max-Planck Institute of Psychiatry, Munich, Germany; 18 Institute of Behavioral Sciences, University of Helsinki, 00014 Helsinki, Finland; 19 Helsinki Collegium for Advanced Studies, University of Helsinki, Helsinki, Finland; 20 Folkhälsan Research Centre, Helsinki, Finland; 21 National Institute for Health and Welfare, Children’s Hospital, Helsinki University Hospital, 00271 Helsinki, Finland; 22 University of Helsinki, 00029 Helsinki, Finland; 23 Department of Obstetrics and Gynecology, MRC Oulu, Oulu University Hospital and University of Oulu, Oulu, Finland; 24 Obstetrics and Gynaecology, University of Helsinki and Helsinki University Hospital, 00014 Helsinki, Finland; 25 Medical and Clinical Genetics, and Obstetrics and Gynecology, University of Helsinki and Helsinki University Hospital, 00014 Helsinki, Finland; 26 Institute for Molecular Medicine Finland, University of Helsinki, 00014 Helsinki, Finland; 27 HUSLAB and Department of Clinical Chemistry, Helsinki University Central Hospital, 00014 Helsinki, Finland; 28 Department of Gynecology and Obstetrics, Emory University School of Medicine, Atlanta, GA US; 29 Department of Obstetrics and Gynecology, University of Texas Medical Branch, Galveston, TX US; 30 Department of Human Genetics, David Geffen School of Medicine University of California Los Angeles, Los Angeles, CA 90095 US; 31 Department of Biostatistics, Fielding School of Public Health, University of California Los Angeles, Los Angeles, CA 90095 US; 32 Department of Psychiatry, University of California, San Francisco, CA US; 33 Department of Pediatrics, University of California, San Francisco, CA US; 34 Department of Preventive Medicine, University of Tennessee Health Science Center, Memphis, TN US

**Keywords:** Developmental age, Aging, Epigenetic clock, DNA methylation, Preterm birth, Cord blood, Fetus, Blood spot, Biomarker, Medicaid, Socioeconomic status, Birthweight

## Abstract

**Background:**

Gestational age is often used as a proxy for developmental maturity by clinicians and researchers alike. DNA methylation has previously been shown to be associated with age and has been used to accurately estimate chronological age in children and adults. In the current study, we examine whether DNA methylation in cord blood can be used to estimate gestational age at birth.

**Results:**

We find that gestational age can be accurately estimated from DNA methylation of neonatal cord blood and blood spot samples. We calculate a DNA methylation gestational age using 148 CpG sites selected through elastic net regression in six training datasets. We evaluate predictive accuracy in nine testing datasets and find that the accuracy of the DNA methylation gestational age is consistent with that of gestational age estimates based on established methods, such as ultrasound. We also find that an increased DNA methylation gestational age relative to clinical gestational age is associated with birthweight independent of gestational age, sex, and ancestry.

**Conclusions:**

DNA methylation can be used to accurately estimate gestational age at or near birth and may provide additional information relevant to developmental stage. Further studies of this predictor are warranted to determine its utility in clinical settings and for research purposes. When clinical estimates are available this measure may increase accuracy in the testing of hypotheses related to developmental age and other early life circumstances.

**Electronic supplementary material:**

The online version of this article (doi:10.1186/s13059-016-1068-z) contains supplementary material, which is available to authorized users.

## Background

Differences in gestational age (GA) as small as one week have been shown to have significant impacts on neonatal morbidity and mortality, as well as long-term outcomes [1–6]. In light of this, the American College of Obstetricians and Gynecologists (ACOG) recently recommended revising the categorization of births from term (>37 weeks gestation) and preterm (≤37 weeks gestation) into several subcategories (early preterm, preterm, early term, full term, late term, and post term) that better reflect the developmental differences associated with GA at each of these time points [7, 8]. Accurate classification systems that reflect both developmental time and maturity may improve our ability to predict neonatal risk.

Traditionally, GA is estimated using one or more of the following methods: early obstetric ultrasound, last menstrual period (LMP), or neonatal estimation [9]. Ultrasound-based methods are considered to be the gold standard and have proven to be a better predictor of delivery date [10] as LMP estimates may be influenced by uncertainty regarding LMP dates, normal variations in ovulation timing, atypical bleeding, and contraceptive use [9]. Neonatal estimation, which is based on a combination of physical appearance, muscular tone, flexibility, and reflexes, is the only available method for determining GA after birth but is less precise than LMP and ultrasound [9, 11, 12]. In circumstances where LMP date is uncertain and ultrasounds are not available, a more accurate method for estimating GA may be beneficial.

Recently, DNA methylation (DNAm) has been used to accurately predict chronological age in children and adults [13–16]. Later work revealed that a methylation-based prediction of age may also associate with physiological consequences in adults when a study reported that an increased methylation age relative to chronological age was associated with an increase in mortality risk [17–22]. However, the predictors optimized in these studies were not designed to estimate GA and did not attempt to differentiate between different GA, as samples taken at birth were either assigned an age of zero or were excluded from the model [13, 14]. Because the accuracy and precision of a prediction model is, in general, weakest at the extremes of the distribution, a predictor developed from primarily adult samples would, by nature, be less accurate in neonates than one that is optimized for that purpose.

DNAm differences in specific CpG sites have been associated with GA at birth in multiple studies [23–26]. We hypothesize that a predictor designed specifically for use with umbilical cord blood or blood spots already routinely collected for newborn screening could allow for accurate neonatal estimation of GA that may also be informative of developmental stage. The objective of this study was to develop such a predictor to estimate GA from DNAm data using umbilical cord blood or blood spot samples and to assess its ability to predict other indicators of developmental maturity.

## Results and Discussion

DNAm data from 1434 neonates, representing 15 independent cohorts, were used for this study. For each sample, HumanMethylation27 or HumanMethylation450 BeadChips (Table 1; Additional file 1: Table S1) were used to generate data from DNA extracted from umbilical cord blood or blood spots. Of the 16,676 CpG sites that passed quality control in the testing and training datasets referenced in Table 1, 3155 (19 %) were at least nominally associated with GA in an epigenome-wide association study (*p* < .05; Additional file 1: Figure S1), and adjustment for proportions of white blood cell subtypes and nucleated red blood cells had little effect on the results (Additional file 1: Figure S2). Associated CpGs were enriched for a range of biological processes, including cell proliferation and chordate embryonic development (Additional file 1: Table S2).

**Table 1:** Description of cohorts

Dataset	N	GA range (weeks)	GA mean ± SD	Male (%)	Race	Nationality	Source	Array
Training datasets								
GSE36642	51	32–38	36.3 ± 1.7	56.9	White	Australian	Cord	27 k
WMHP1	40	31–41	37.9 ± 2.3	47.5	98 % white	American	Cord	450 k
GSE62924	38	34–41	39.1 ± 1.4	42.1	White	Mexican	Cord	450 k
NBC	36	24–41	36.0 ± 5.4	47.2	Black	American	Cord	450 k
GSE51180	23	25–42	32.7 ± 6.6	69.6	White	Australian	Spot	450 k
GSE30870	19	34–41	38.9 ± 2.1	NA	White	Spanish	Cord	450 k
Test datasets								
DNSBtrios	264	28–44	40.3 ± 1.9	64.9	White	Danish	Spot	450 k
WMHP2	251	33–43	38.7 ± 1.4	51.0	80 % white	American	Cord	27 k
CANDLE	198	32–41	39 ± 1.3	52.0	51 % black, 40.4 % white	American	Cord	27 k
VICS	183	24–35	28.0 ± 2.1	42.1	89 % white	Australian	Spot	450 k
PROGRESS	148	30–43	38.6 ± 1.7	52.0	White	Mexican	Cord	450 k
PREDO	91	31–42	39.6 ± 1.5	54.9	White	Finnish	Cord	450 k

Training datasets and test datasets were chosen to represent a similar range of gestational ages

*NA* not available, *SD* standard deviation

### Predicting DNAm GA in neonates

**Fig. 1:**
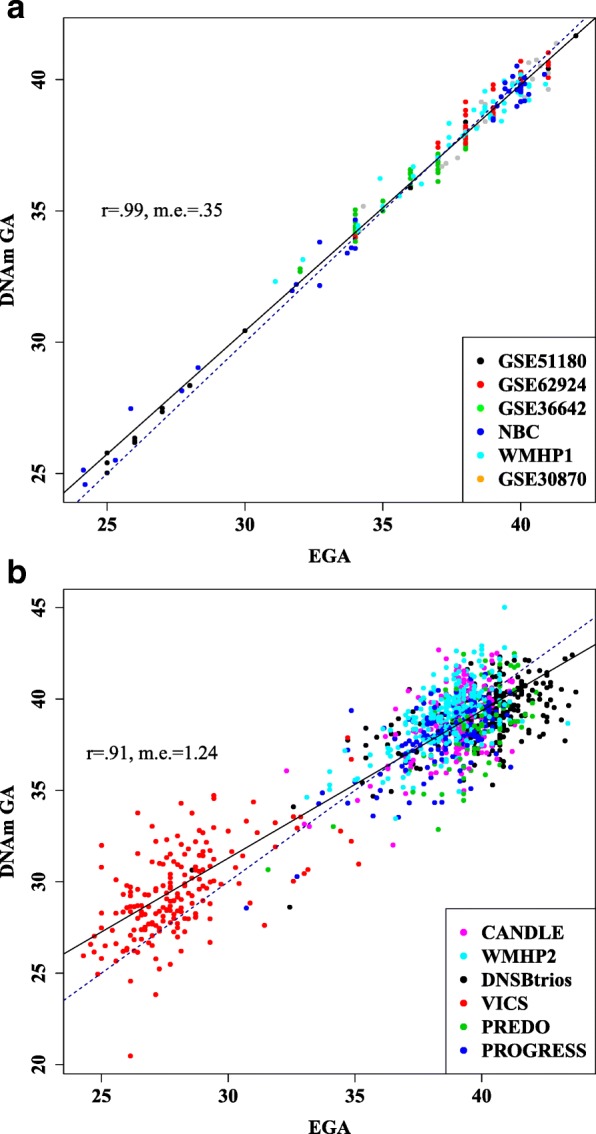
Correlation between DNAm GA and GA. **a** DNAm GA and estimated clinical GA (*EGA*) are highly correlated in the training dataset: r = 0.99, median error (*m.e.*) = 0.35. **b** DNAm GA and estimated clinical GA were also highly correlated in the testing dataset: r = 0.91, median error = 1.24. *Solid line* = regression line; *dotted line* indicates equivalence

To train the DNAm GA predictor, six independent cohorts were selected to sample a wide range of GAs and ancestries. Consistent with the approach described by Horvath [13], elastic net regression was used to select a set of 148 CpG sites (Additional file 2) predictive of GA from a set of 16,838 CpG sites that were available in all training datasets. Although some of the individual studies report associations between the perinatal environment and DNAm, no CpG site reported to associate with environmental exposures in these cohorts were among the sites selected for this predictor [27–30]. Overall, 90 out of 148 CpG sites selected for the predictor (61 %) showed some evidence for association with GA in the cell type-adjusted epigenome-wide association study (*p* < 0.05). In the training datasets, correlation between the resulting predictor (DNAm GA) and clinically estimated GA was 0.99 (Fig. 1a), indicating a strong fit of the model. The 148 CpG sites selected by the elastic net were uniformly distributed across the genome and were not located in genes more likely to be represented among specific biological pathways (data not shown). They were more likely to reside in CpG island shores than the remaining 16,690 CpG sites that were eligible for inclusion in the predictor (odds ratio (OR) = 1.73; *p* = 0.00096) and less likely to reside in CpG islands (OR = 0.53; *p* = 0.00019) or active promoters (OR = 0.59; *p* = 0.0028). The 148 sites showed no significant enrichment or depletion for CpG island shelves or enhancers (Additional file 1: Table S3). They were also not enriched or depleted for sites with genetic variants located in the probe sequence or sites previously reported to associate with African American or Caucasian race (Additional file 1: Table S3) [31–33].

The predictive accuracy of the model was tested in 1135 samples from six independent datasets. The testing and training datasets had comparable GA distributions (Additional file 1: Figure S3). In the testing datasets, overall correlation between DNAm GA and GA was 0.91 (*p* < 2.20 × 10^−16^; Fig. 1b). Within individual test datasets, correlation between GA and DNAm GA remained high (0.52 < r < 0.65; Additional file 1: Figure S4) though appeared lower than in the combined dataset due to lower sample sizes and GA range. We were not able to obtain similar predictive power using the DNAm age predictor proposed by Horvath, which has a highly significant but much weaker correlation with GA (r = 0.14, *p* = 4.89 × 10^−6^; Additional file 1: Figure S5). This correlation coefficient is similar to that observed for prenatal brain samples (r = 0.15) [34].

We did not evaluate the Hannum predictor [14] since it is less accurate than the Horvath predictor in children [14, 35]. Of note, only six CpG sites included in the DNAm GA predictor overlap with CpG sites in the predictor designed by Horvath and no sites overlap with the predictor designed by Hannum. However, one would not necessarily expect overlap. Elastic net regression selects a parsimonious set of the full list of CpG sites and among highly correlated CpG sites only one may be chosen, introducing an element of chance into CpG selection. Moreover, the late gestational period is associated with unique developmental changes that cannot be discriminated by the adult predictor, which did not include measures of GA in its training dataset. Thus, this lack of overlap may indicate that the CpG sites predictive of GA in neonates are distinct from CpG sites predicting age in adults because of their association with changes specific to gestational development.

The average absolute difference between DNAm GA and GA in test samples was 1.49 weeks, with a standard deviation of 1.16 weeks. The median absolute difference (“median error”) between DNAm GA and GA was 1.24 weeks. This falls well within the range of error for clinical estimates of GA based on either LMP or ultrasound, as each of these clinical measures has an inherent variability due to recall bias and natural phenotypic variation associated with development [9, 10, 36]. However, it was interesting to note that DNAm GA correlated more strongly with clinical GA estimates based on ultrasound than those based exclusively on LMP (Additional file 1: Figure S6). Error rates for ultrasound range from 5–7 days if performed during the first trimester to 3.0–4.3 weeks when performed in the third trimester. This predictor is closer to clinical estimates of GA than post-birth measures using neonatal estimation, which can overestimate the GA of preterm neonates by up to 2.57 weeks [37–41].

The accuracy of this predictor is consistent with that of established clinical methods for estimating GA, though its accuracy can only be interpreted in the context of the available clinical measurements. Predictive accuracy was not influenced by neonatal sex as there was no difference between the median errors in males versus females (*p* = 0.76). The median error between DNAm GA and clinically estimated GA was 1.07 for the cord blood datasets and 1.57 for blood spot datasets. This discrepancy may be due to differences in the precision of GA, as sample collection for blood spots was performed up to 39 days after birth (Additional file 1: Figures S3 and S7). It is also possible that there may be differences in sample quality, as some blood spot samples were stored for more than 30 years, although there was no difference in the number of samples that failed quality control between the cord blood and blood spot datasets. The correlation between DNAm GA and clinically estimated GA was 0.94 (median error (m.e.) = 1.4) for samples processed on the HumanMethylation450 array and 0.55 (m.e. = 1.02) for samples processed on the HumanMethylation27 array (Additional file 1: Figure S8). This discrepancy is likely due to the differences in GA range between samples run on the two arrays (19.3 and 11.1 weeks, respectively). Finally, the partial correlation from regressions of DNAm GA on clinically estimated GA did not substantially change when cell composition covariates were included, suggesting that the accuracy of the predictor is not confounded by cellular heterogeneity (r_original_ = 0.91, r_cell type adjusted_ = 0.81).

To limit concerns regarding the potential for overfitting of the models, we next validated the predictor in a second testing dataset, comprised of 92 samples from three cohorts (FAP, GSE66459, and GSE69633) that were not included in the initial testing or training sets. Cohort demographics are provided in Additional file 1: Table S3. The correlation in these datasets is similar to that of the first testing dataset (r = 0.89, m.e. = 0.89; Additional file 1: Figure S9), further indicating that this model fits well when applied to novel datasets and should be generalizable to other studies.

### Accuracy of DNAm GA in the same subjects

**Fig. 2:**
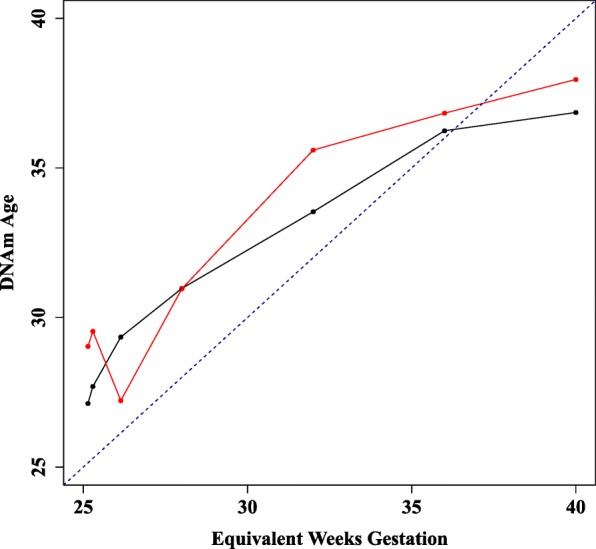
Reproducibility of DNAm GA. DNAm GA from birth until term equivalency for two subjects recruited through the EpiPrem study, gestational age at birth 25 weeks. DNAm GA increases appropriately with gestational age in weeks. Change in DNAm GA over equivalent weeks gestation

Serial blood sampling was conducted from two neonates admitted to the Neonatal Intensive Care Unit (NICU), independent of the testing and training samples. Seven timepoints were collected between birth at 25 weeks and discharge at 40 weeks. DNAm GA increased as expected over time from birth until term equivalency (Fig. 2). These pilot data demonstrate that the predictor has the sensitivity required to detect changes in DNAm GA in the magnitude of days or weeks and that methylation patterns change from birth to term equivalency in a predictable manner.

### DNAm GA as a measure of developmental age

In adults, the difference between DNAm-based age estimates and chronological age associates with all cause mortality, HIV status, and Down syndrome [17, 42, 43]. This difference is usually described as age acceleration [13]. We calculated a similar measure, which we will subsequently refer to as “GA acceleration”, in our cohorts by using the residual of a linear model regressing DNAm GA on clinically estimated GA. Because accelerated GA may indicate increased developmental maturity, we sought to evaluate whether GA acceleration associated with perinatal measures of health and development in the cohorts with available data.

**Fig. 3:**
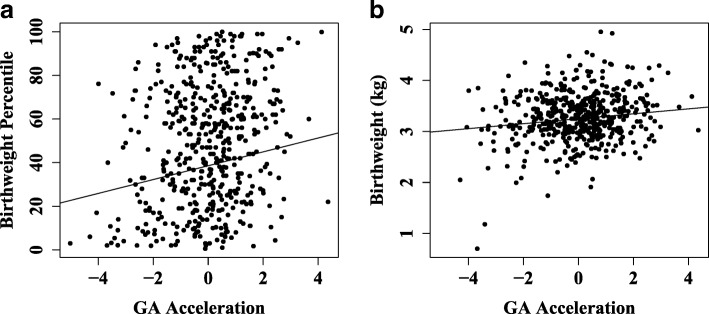
GA acceleration associates with birthweight. The association between GA acceleration and **a** birthweight percentile (*p* = 4.5 × 10^−4^) or **b** birthweight (*p* = 0.033) adjusted for race, cellular composition, cohort, and gestational age in CANDLE, WMHP, and PROGRESS. *Solid line* = regression line

Birthweight is widely used as a proxy of developmental maturity in studies assessing the association between the prenatal environment and short-term or long-term neonatal risk, with those born at the lowest birthweight generally having the highest risk for mortality over the first year of life and for cardio-metabolic conditions as adults [44, 45]. Birthweight is positively correlated with GA so birthweight percentile, which is calculated based on birthweight averages for a given GA corrected for fetal sex, is commonly used as an indicator of perinatal health [46, 47]. Previous studies have shown that infants in the lowest birthweight percentiles have an increased risk of perinatal death and other adverse outcomes and are often defined as growth restricted [48, 49]. In cohorts with available data, GA acceleration significantly predicted birthweight percentile (*p* = 4.5 × 10^−4^; Fig. 3a) and birthweight (*p* = 0.033; Fig. 3b) after correcting for clinically estimated GA, race, estimated cell type proportions, and cohort. Consistent with the idea that DNAm GA may reflect maturity, the fitted regression model predicts approximately the 50th percentile to have GA acceleration of 0. Thus, neonates falling in the lowest birthweight percentiles show lower, while neonates falling in the highest percentiles show higher or accelerated GA. There was no association between GA acceleration calculated using the DNAm age predictor of Horvath [13] and either birthweight or birthweight percentile (Additional file 1: Figure S10).

**Fig. 4:**
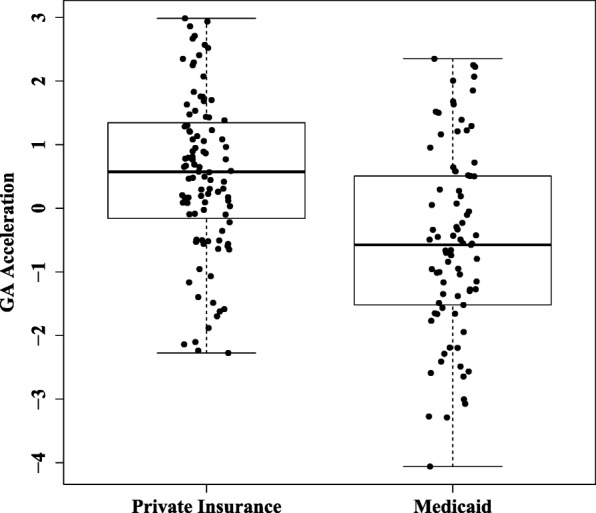
Maternal insurance status and GA acceleration. Neonates born to mothers with private insurance have higher GA acceleration than neonates born to mothers on Medicaid (*p* = 0.023) after adjusting for race, gestational age, and cellular composition

One study by Appleton and colleagues [50] suggests that socioeconomic adversity promotes adverse health outcomes through epigenetic programming of neonatal DNAm. We hypothesize that factors related to early life adversity might influence the developmental age of the neonate. One such factor is socioeconomic status, which is essential to examine as children born into socioeconomically disadvantaged families, often operationalized by insurance status (Medicaid versus private health insurance), have poorer health in childhood and early adulthood [51, 52]. In the most socioeconomically diverse cohort (CANDLE), GA acceleration associated with maternal Medicaid status (*p* = 0.023) after adjusting for race, clinically estimated GA, and estimated cell type proportions (Fig. 4). Specifically, methylation-based estimates of GA were lower than clinical estimates for the neonates of women on Medicaid compared with women with private health insurance. This association supports the hypothesis that prenatal adversity associates with changes in neonatal methylation consistent with a delayed developmental age, which may have consequences later in life.

## Conclusions

GA can be accurately predicted between 24 and 44 weeks gestation using DNAm values obtained from both umbilical cord blood and blood spot samples. DNAm GA is more concordant with GA estimates performed with the gold standard of ultrasound than with estimates based on LMP. However, the question remains as to whether GA acceleration is truly a measure of maturity versus a reflection of the relative accuracy of DNAm GA compared with clinical estimates. We consider three possibilities for interpreting the difference between DNAm GA and clinically estimated GA. First, an accelerated GA may reflect differences in physiological development of the neonate such that neonates with a higher DNAm GA are more developmentally mature than their chronological age suggests. A second possibility is that the differences between DNAm GA and chronological GA reflect epigenetic programming by early life environmental exposures, such as maternal prenatal stress or pregnancy disorders, which may affect neonatal outcomes and development [53]. Finally, any difference may simply be reflective of the variable nature of clinical GA estimations; evaluation of DNAm GA in neonates conceived through in vitro fertilization would be helpful for delineating these different possibilities. These models may be interrelated, such that the true interpretation is likely a combination of these possibilities. A future study examining other prediction methods, including the use of non-linear models or transformations, may facilitate this interpretation by further delineating developmental differences between early and late GAs. Overall, our results suggest that DNAm GA and GA acceleration are promising tools for evaluating neonatal developmental maturity.

A targeted assay of the CpG sites necessary to compute DNAm GA could provide a rapid and robust estimator of GA at birth, and the framework described in this paper could be used to develop and validate a predictor based on other tissues that may be sampled prior to delivery, such as chorionic villi or amniotic fluid. Our results suggest that DNAm GA is highly reproducible and can predict measures of developmental maturity, such as birthweight, better than clinical estimates of GA alone. As such, it has the potential to serve as a biomarker for GA and the rate of neonatal development. Recent studies of GA and DNAm [23–26] support that shifts in methylation underlie the aging process, further supporting the development of methylation-based biomarkers. DNAm is a convenient molecular marker for GA in that umbilical cord blood and blood sampling are routinely performed to monitor neonatal health in humans, and it can be readily sampled repeatedly in the same person, as demonstrated by the time course data in the subjects from preterm birth through term equivalency.

As a biomarker, DNAm GA and GA acceleration would have numerous clinical, research, and forensic applications. It would serve as a molecular marker of GA that complements clinical estimates, when available, and provides additional information when clinical estimates are unavailable or unreliable. For example, it could be used to estimate GA in women who seek prenatal care late in pregnancy, are unsure of the date of their last menstrual period, or did not have ultrasounds performed early in pregnancy. DNAm GA is more precise than the estimation methods typically performed at birth, which rely on biometric measurements. Precise knowledge of GA would be most informative for neonates born extremely preterm, when parents and clinicians are confronted with decisions regarding active intensive care interventions versus providing comfort care. GA based on an epigenetic developmental profile may also complement clinical estimates of GA, providing a screening tool to identify children who may benefit from additional monitoring and care. Studies to explore the extent to which DNAm GA reflects developmental maturity, and thus may be a more reliable predictor of outcomes after preterm birth compared to time or growth-based methods, are needed.

DNAm GA may also serve as a surrogate marker for developmental maturity in research studies of neonatal development, interventions, and disease. Our results already demonstrate that it will be fruitful to study antenatal and perinatal factors that associate with DNAm GA and GA acceleration and to determine whether these metrics are better prognosticators of neonatal well-being than conventional measures. Future studies should evaluate the effects of maternal stress, nutrition, and interventions such as vitamin supplementation that are highly relevant to fetal development and pregnancy outcomes. Future research could also explore whether GA acceleration relates to risk of developing pediatric disorders, such as autism, and whether it can predict health outcomes later in life. Finally, establishing precise GA is important for forensic, anthropologic, or other medico-legal investigations. Indeed, DNAm-based predictors of adult age are already under investigation for forensic applications [54]. In summary, we have identified a potential biomarker for GA with an abundance of applications that warrant further investigation and development.

## Methods

### Description of cohorts

Training datasets were selected to include a wide range of GAs and ancestries. Publically available datasets were downloaded from the Gene Expression Omnibus (GEO): GSE36642, GSE62924 [27], GSE51180 [55], and GSE30870 [56]. Methylation data for all of these datasets were generated on either the Illumina Infinium HumanMethylation27 BeadChip or Infinium HumanMethylation450 BeadChip (Table 1). These methods have been shown to be highly reproducible and consistent with the results of other epigenetic methods [57, 58]. For umbilical cord blood samples, GA was defined as the GA at birth. For blood spot samples, GA was defined as the GA plus the number of days that occurred between birth and sampling. The individual cohorts are detailed in Additional file 1.

### Quality control and normalization

All analyses were performed using R version 3.1.2. Datasets used in this study underwent several quality control measures. The DNAm age predictor developed by Horvath was initially run on all samples to establish predicted age and gender [13]. Samples with gender discordance or estimated age >1.5 years were excluded from further analysis. After this initial quality control step, datasets were subjected to standard quality control through the use of the R package CpGassoc [59]. A data frame consisting of β values (Methylated signal/(Methylated signal + Unmethylated signal)) was supplied as input to CpGassoc. Any data point with a detection *p* value above 0.001 was set to missing. CpG sites with >5 % missing data were excluded; subsequently, samples with >5 % missing data were excluded. These quality control measures were performed to ensure that the predictor is built based on high quality probes and samples. Any probe missing entirely from one of the datasets was excluded from the remaining datasets, so only probes passing quality control in all training datasets and probes present on both the HumanMethylation450 and HumanMethylation27 arrays were included, for a total of 16,838 probes. Finally, datasets were normalized according to Horvath’s modified beta-mixture quantile (BMIQ) normalization [13, 60]. While the original BMIQ is a within-sample normalization method to address probe type bias by modifying the type II distribution to match that of type I probes, Horvath modified this BMIQ procedure for a different purpose: the distribution of each given array is related to that of a “gold standard” array (defined here as the mean across all of the training datasets). Thus, Horvath’s modification of the BMIQ method could be interpreted as a form of between-sample normalization. All training datasets were normalized together, as a single group. After normalization, missing values for each sample were imputed by the *k*-nearest neighbors method where *k* = 10, using the R package impute so that no missing values remain in the dataset after pipeline completion [61]. Test datasets were normalized separately, following the same procedures as above. One test cohort, PROGRESS, which was processed with an out of band background correction, dye bias correction, and then the original BMIQ procedure, was excluded from the quality control pipeline as raw files were not available. Principal components analysis was used to assess the potential impact of BeadChip on the CpG sites selected for inclusion in the predictor. We did not observe clustering by chip (Additional file 1: Figure S11), suggesting that the chip was not a confounding factor.

### Estimation of cellular composition

Proportions of white blood cells and nucleated red blood cells were estimated from genome-wide DNAm patterns using the method proposed by Houseman et al. [62], with reference samples from homogenous cell populations for white blood cells (CD4^+^ T cells, CD8^+^ T cells, natural killer cells, B cells, monocytes, and granulocytes), nucleated red blood cells [63, 64], and whole blood (GSE80310).

### Epigenome-wide association study

The R package CpGassoc [59] was used to perform epigenome-wide association studies (EWAS) to assess associations between GA and DNAm. Two separate EWAS were performed, with and without the inclusion of cellular composition covariates. Each EWAS was performed as a meta-analysis across all cohorts by including indicators for each study as covariates. Test statistics from the two EWAS were plotted to assess the robustness of results to potential cell type heterogeneity.

### Elastic net regression and age prediction

The six training datasets (GSE36642, WMHP1, GSE62924, NBC, GSE51180, and GSE30870) were combined to perform an elastic net regression of GA on the 16,838 CpG probes remaining after quality control and filtering. The regression was performed using the R package glmnet to select a parsimonious set of CpG sites predictive of GA. Following Horvath [13], the elastic net mixing parameter, alpha, was set to 0.5 allowing for equal contribution of the ridge and lasso methods [65]. The lambda parameter was chosen through a tenfold cross validation, which involves randomly partitioning the training dataset into ten equally sized subsamples. The cross-validation procedure is then performed ten times, retaining a different subsample as a validation dataset each time. In the procedure, data from the other nine subsamples are used to build a predictor based on a particular value of lambda, and the fit of the predictor is then tested in the omitted validation set. The mean squared error is calculated for the validation set in each iteration and then averaged over the ten subsamples. This procedure is performed for a sequence of lambda values to determine the lambda that yields the minimum mean squared error. No additional covariates were included in the analysis, consistent with the development of the DNAm age predictor by Horvath [13]. The training coefficient values and CpG probes selected from this regression were used to fit a linear model to generate predicted values of GA, based on a modified version of the R code in the DNAm age tutorial published by Horvath [13]. The accuracy of predicted values of GA was determined from correlation coefficients obtained through linear regression of DNAm GA and clinical GA.

### Analysis of GA acceleration

GA acceleration was calculated as the residual from a linear regression of DNAm GA on clinical estimates of GA for the combined testing dataset. Analysis of DNAm GA with birthweight and birthweight percentile was then conducted using linear regression of birthweight and birthweight percentile on GA acceleration and covariates for race, estimated cell type proportions, and cohort. Clinically estimated GA was included as a covariate in the analysis for birthweight but not birthweight percentile as birthweight percentile is already adjusted for clinically estimated GA. Maternal insurance status (as a proxy for income) was analyzed in the CANDLE cohort through logistic regression of maternal insurance status on GA acceleration, adjusting for estimated clinical GA, race (African American versus Caucasian), and estimated cell type proportions.

### Enrichment tests

To assess whether the CpG sites selected for the DNAm GA predictor were more likely than others to be located in functionally relevant regions, two approaches were used. First, CpG positions were intersected with the hg19 CpG island annotation track from the UCSC Genome Browser (http://genome.ucsc.edu) to define whether each site was located in a CpG island, CpG shore (±1.5 kb from island), or CpG shelf (±1.5 kb from shore). Second, the CpG positions were intersected with ENCODE’s ChromHMM annotation for lymphoblastoid cell line GM12878, which uses a hidden Markov model to assign genomic features based on the combinatorial pattern of various chromatin marks [66]. The ChromHMM annotation allowed identification of CpGs located in promoters and enhancers. Fisher’s exact test was used to assess whether there was significant enrichment of each feature in CpG sites selected for the predictor compared to the full set of 16,838 sites included in the elastic net model. A similar analysis was preformed to assess whether these CpG sites were enriched for sites containing a genetic variant in the 50-bp probe (using annotation derived from the 1000 Genomes Project) or sites previously reported to associate with race [31]. DAVID was used to evaluate whether CpG sites used to estimate DNAm GA were located in genes enriched for any biological pathways [32].

## Additional files

Additional file 1: Figures S1–S11 and **Tables S1–S3.** (PDF 3090 kb)
Additional file 2: CpG sites and corresponding genes used to predict DNAm age. (XLSX 43 kb)
Additional file 3: CpG sites and coefficients used to predict DNAm age. (CSV 3 kb)
Additional file 4: Wrapper program for predicting gestational age. (R 6 kb)
Additional file 5: R code to normalized and predict DNAm age. (R 6 kb)
Additional file 6: Test dataset for predicting DNAm age. (CSV 1240 kb)
Additional file 7: Instructions for predicting gestational age from cord blood and blood spot methylation. (DOCX 137 kb)
Additional file 8: Relevant data from PROGRESS cohort. (CSV 282 kb)
