# Supplementary material for: An epigenetic clock for gestational age at birth based on blood methylation data
Source: Genome Biol. 2016 Oct 7;17:206. doi: 10.1186/s13059-016-1068-z (PMC5054584; doi:10.1186/s13059-016-1068-z)
Supplement: Additional file 1: Figures S1–S11 — and Tables S1–S3. (PDF 3090 kb) [file 13059_2016_1068_MOESM1_ESM.pdf]

Supplementary Tables & Figures (pp. 1-14), followed by descriptions and methods for each cohort (pp. 15-21)

Table S1: Description of additional testing cohorts. Additional cohorts were identified to further validate the accuracy of the predictor.

Testing Datasets							
**Dataset**	**N**	**GA Range**	**GA mean ± SD**	**%Male**	**Race**	**Nationality**	**Source**
**FAP**	24	38-43	40.1±1.2	45.8	83% White	American	Cord
**GSE66459**	22	26-42	35.2±5.2	50.0	White	Dutch	Cord
**GSE69633**	46	36-41	38.9±1.3	50.0	White	Mexican	Cord

Figure S1: Manhattan plot showing the distribution of GA-associated CpG sites across the genome. Points falling above the dashed horizontal line indicate experiment-wide significance FDR<.05; points above the solid horizontal line are significant according to a more conservative step-down Bonferroni adjustment for 16,676 CpG sites.

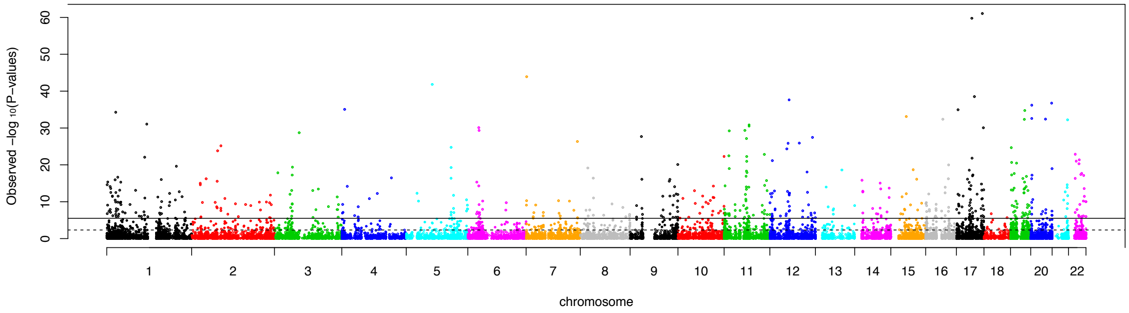

Figure S2: Comparison of t-statistics from two epigenome-wide associations studies (EWAS) of gestational age to assess robustness of results to cell type heterogeneity. The x-axis shows t-statistics from an EWAS for gestational age adjusting for estimated cellular composition (proportions of six white blood cell subtypes and nucleated red blood cell counts) while the y-axis shows t-statistics unadjusted for cell composition.

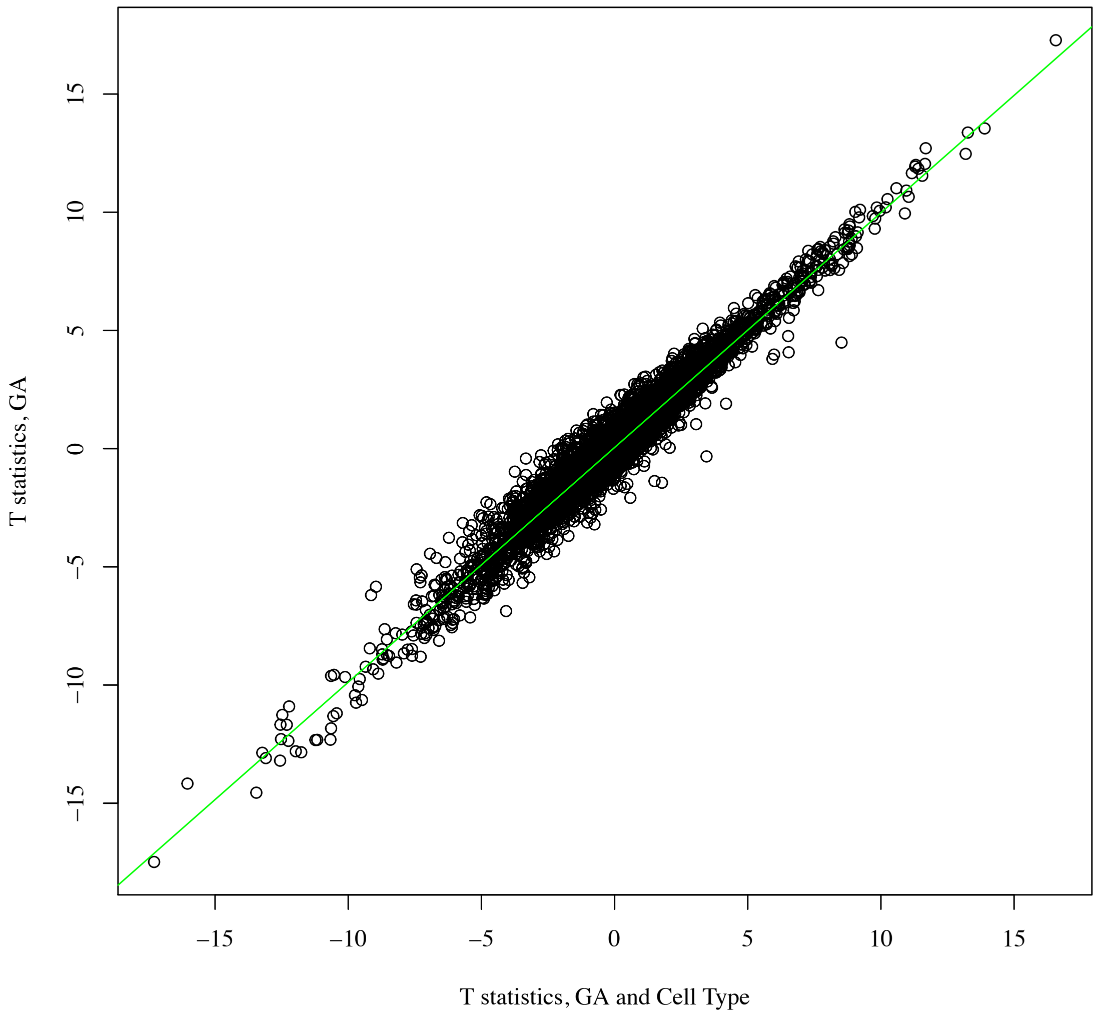

Table S2: Enrichment for the top 20 biological processes in genes containing GA-associated CpG sites. The p-value is adjusted using a Benjamini- Hochberg correction.

Biological Process	Count	%	p-value
Biological adhesion	172	6.3	7.60E-07
Cell adhesion	172	6.3	1.40E-06
Regulation of cell proliferation	184	6.7	6.60E-06
Regulation of phosphorylation	116	4.2	1.00E-04
Cell motion	118	4.3	1.00E-04
Regulation of phosphorus metabolic process	120	4.4	1.20E-04
Regulation of phosphate metabolic process	120	4.4	1.20E-04
Cell migration	78	2.8	1.20E-04
Cell motility	83	3	1.80E-04
Localization of cell	83	3	1.80E-04
Vasculature development	71	2.6	1.90E-04
Intracellular signaling cascade	260	9.5	1.90E-04
Chemical homeostasis	123	4.5	2.00E-04
Regulation of cell motion	58	2.1	2.50E-04
Blood vessel development	69	2.5	2.50E-04
Regulation of cell adhesion	45	1.6	3.30E-04
Positive regulation of cell proliferation	102	3.7	4.70E-04
Regulation of cell death	177	6.4	5.20E-04
Chordate embryonic development	85	3.1	6.10E-04
Cell activation	76	2.8	6.30E-04

Table S3: Enrichment tests for 148 CpG sites selected by elastic net regression. Significant enrichment (OR > 1) or depletion (OR < 1) after adjustment for 12 tests (p < .05/12) is indicated by **bold text**.

**Enrichment/depletion for:**	**OR**	**95% C.I.**	**p-value**
CpG islands	0.53	(0.37, 0.75)	**.00019**
CpG island shores	1.73	(1.24, 2.43)	**.00096**
CpG island shelves	0.76	(0.34, 1.49)	.54
Promoter regions	0.70	(0.50, 0.99)	.038
- Active promoters	0.59	(0.41, 0.84)	.**0028**
- Weak promoters	1.26	(0.74, 2.03)	.36
- Poised promoters	1.26	(0.65, 2.24)	.42
Enhancer regions	1.38	(0.76, 2.33)	.23
- Strong enhancers	1.79	(0.75, 3.65)	.14
- Weak enhancers	1.09	(0.46, 2.21)	.71
Race-associated CpG sites	0.97	(0.57, 1.57)	.99
CpG sites with genetic variants in probe	0.78	(0.45, 1.27)	.37

OR: odds ratio.
C. I.: confidence interval

Figure S3: Distribution of clinically estimated gestational age (EGA) ranges in the training (A) and testing (B) datasets.

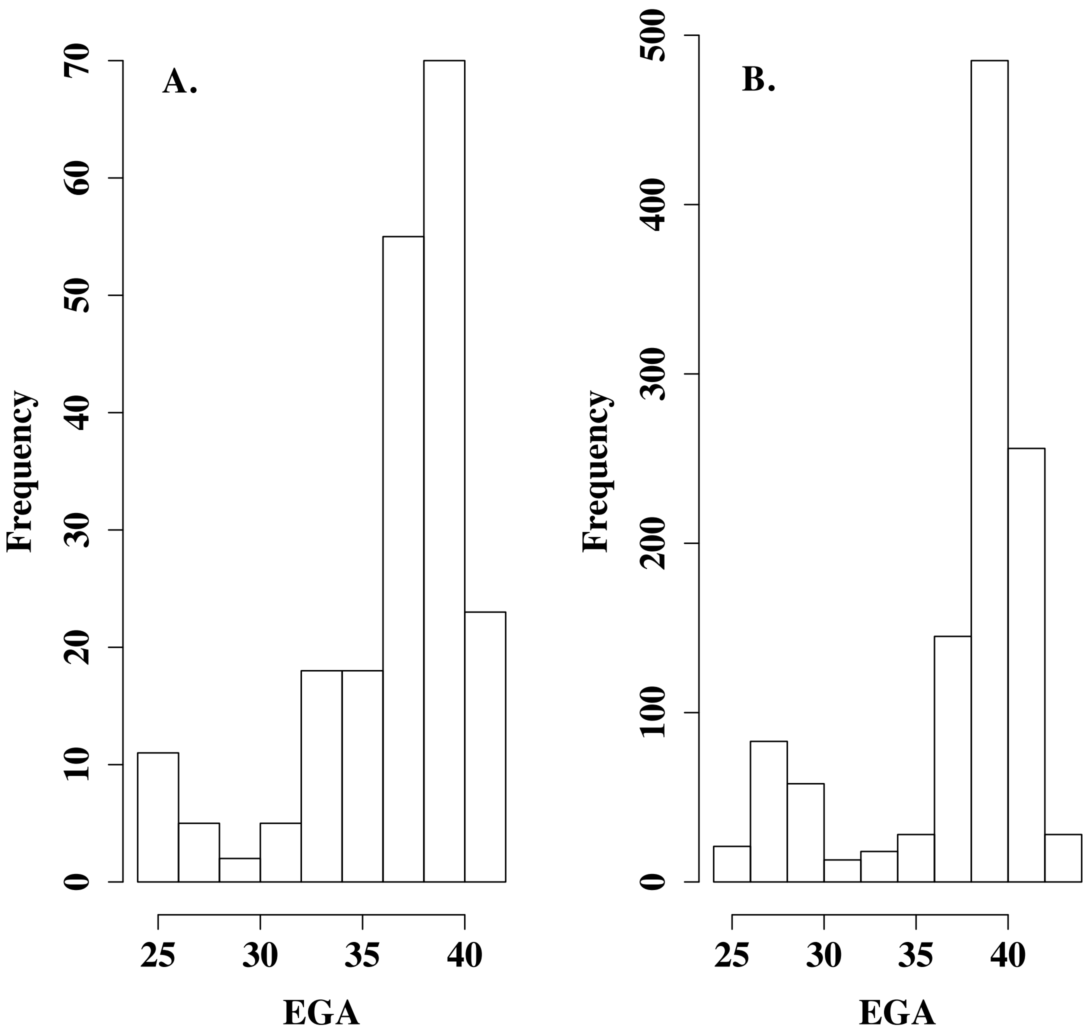

Figure S4: Correlation between clinically estimated gestational age (EGA) and DNAm GA for each testing dataset. Solid line = regression line; dotted line indicates equivalence. Median absolute difference (‘median error’) = m.e.

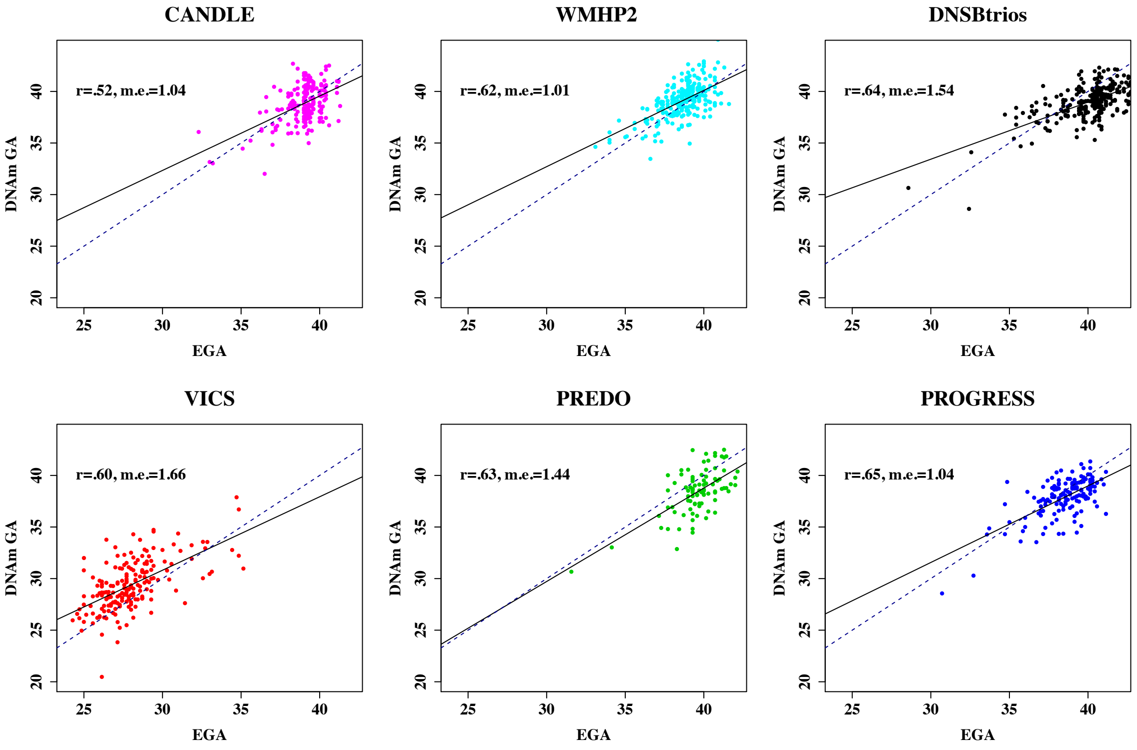

Figure S5: Correlation between clinically estimated GA (EGA) and DNAm age estimated using the Horvath predictor [2] (r=.14, p= 4.89×10^{-6}, median error=9.19). DNAm age is represented in equivalent weeks gestation. Red= VICS, fuchsia= CANDLE, cyan= WMHP2, black= DNSBtrios, green= PREDO, blue= PROGRESS. Solid line = regression line; dotted line indicates equivalence.

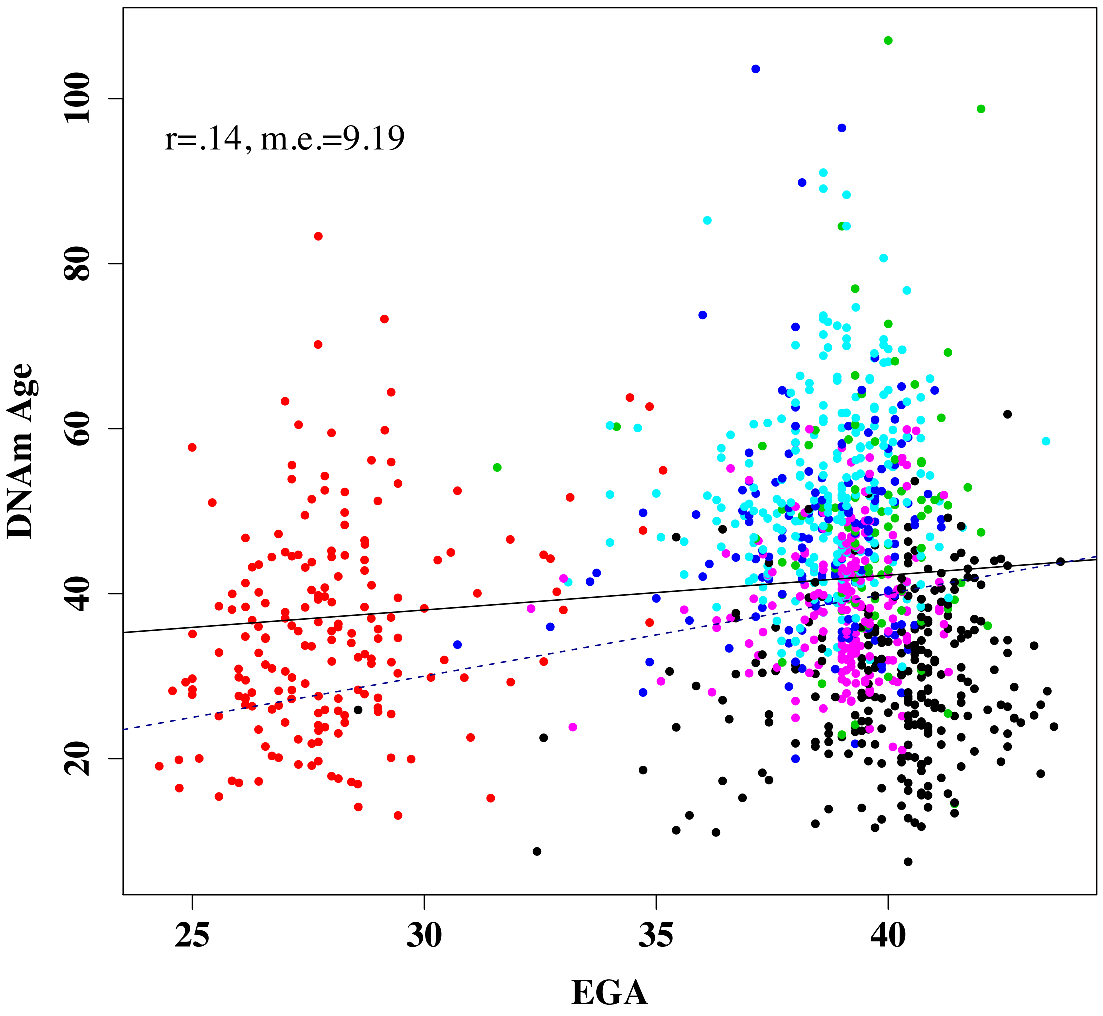

Figure S6: Comparison of DNAm GA with clinical GA estimates based on LMP (last menstrual period) or ultrasound in WMHP2. (r_{LMP}=.41, p_{LMP}= 5.5 × 10^{-8}, median $error_{LMP}$=.93; $r_{Ultrasound}$=.54, $p_{Ultrasound}$= 4.79 × 10^{-14}, median $error_{Ultrasound}$=1.01).

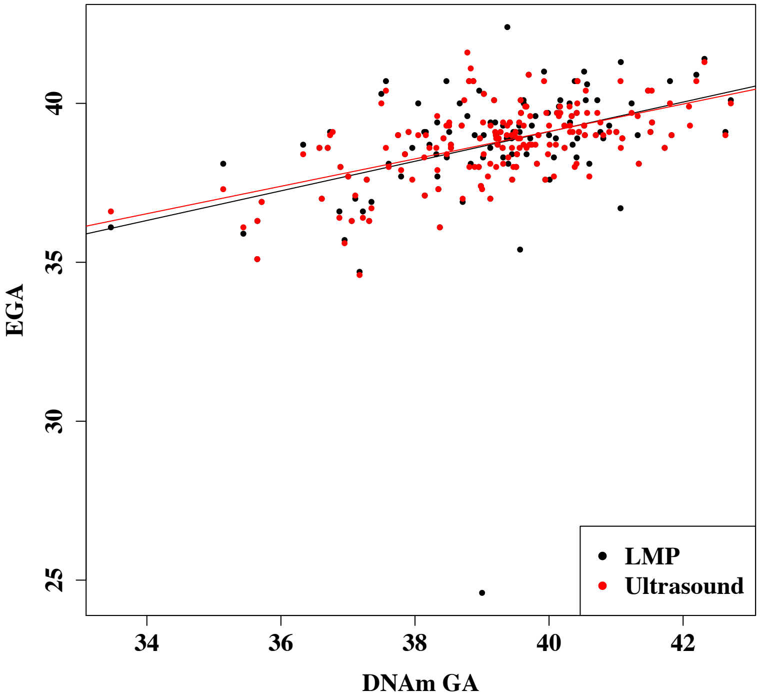

Figure S7: Predictive accuracy of DNAm GA in cord blood and blood spot samples. Clinically estimated GA (EGA) is depicted on the x-axis. $r_{CordBlood}$=.57, $p_{CordBlood} < 2.2 \times 10^{-16}$, median $error_{CordBlood}$=1.57; $r_{BloodSpot}$=.95, $p_{BloodSpot}$ $p < 2.2 \times 10^{-16}$, median $error_{Ultrasound}$=1.07

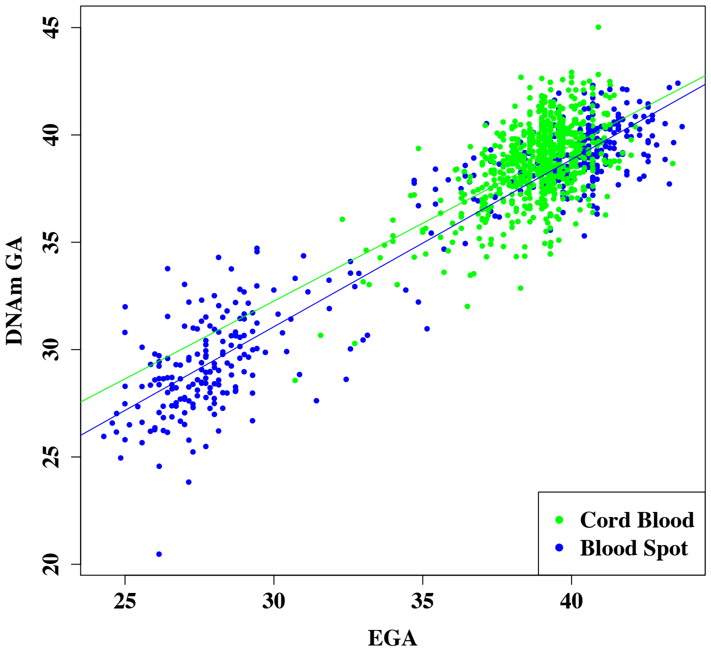

Figure S8: Predictive accuracy of DNAm GA on the HumanMethylation27 array (A) and HumanMethylation450 array (B).

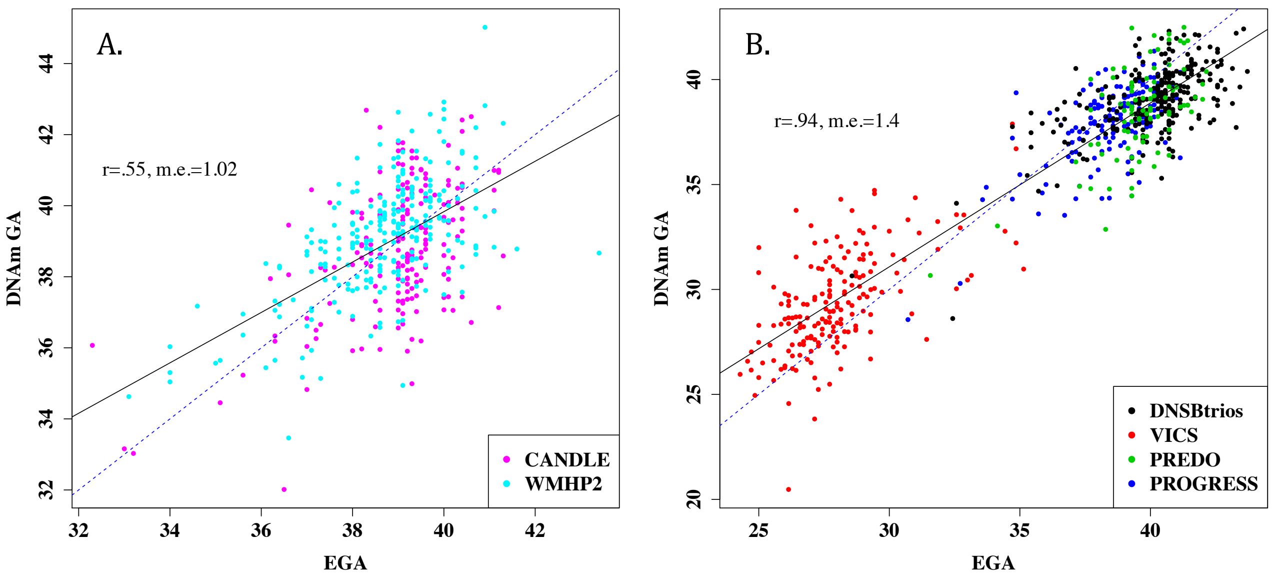

Figure S9: Predictive accuracy of DNAm GA on three additional testing datasets (FAP, GSE66459, GSE69633).

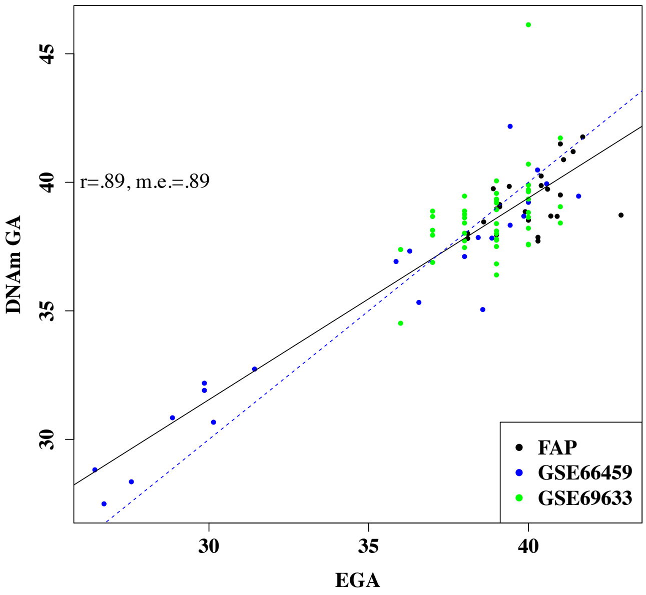

Figure S10: Association between birthweight percentile (A) and birthweight (B) and age acceleration calculated using the DNAm age predictor [2]. $r_{birthweight}$= .0014, $p_{birthweight}$ = .79; $r_{birthweightpercentile}$= .05, $p_{birthweightpercentile=}$ .28 Solid line = regression line.

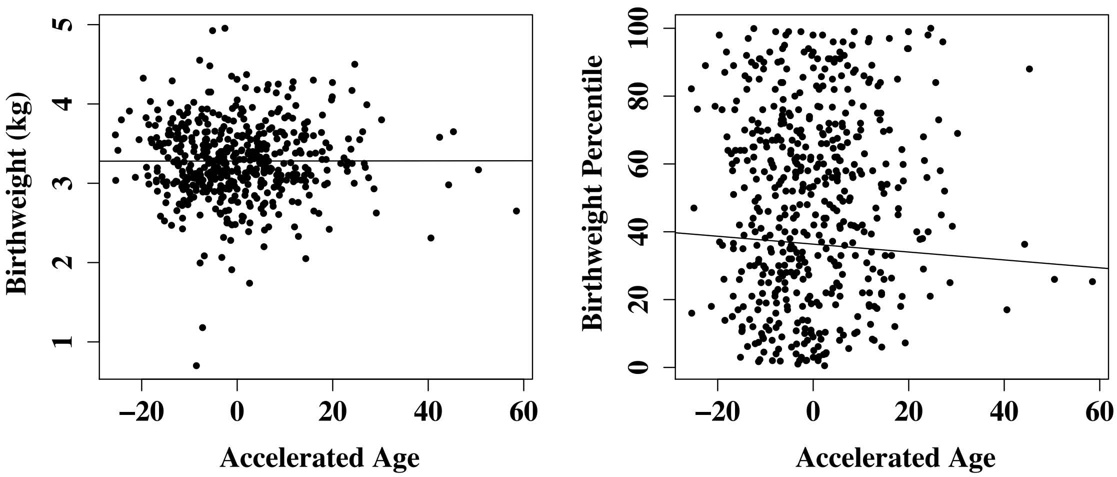

Figure S11: Plots for the first two principal components, colored by chip, of the 148 CpG sites included in the predictor in the training (GSE62924, NBC, WMHP1) and testing datasets (CANDLE, DNSBtrios, PREDO, PROGRESS, VICS, and WMHP2) for which chip/batch information was available. Note that GSE62924 was colored by batch as the chip information was unavailable.

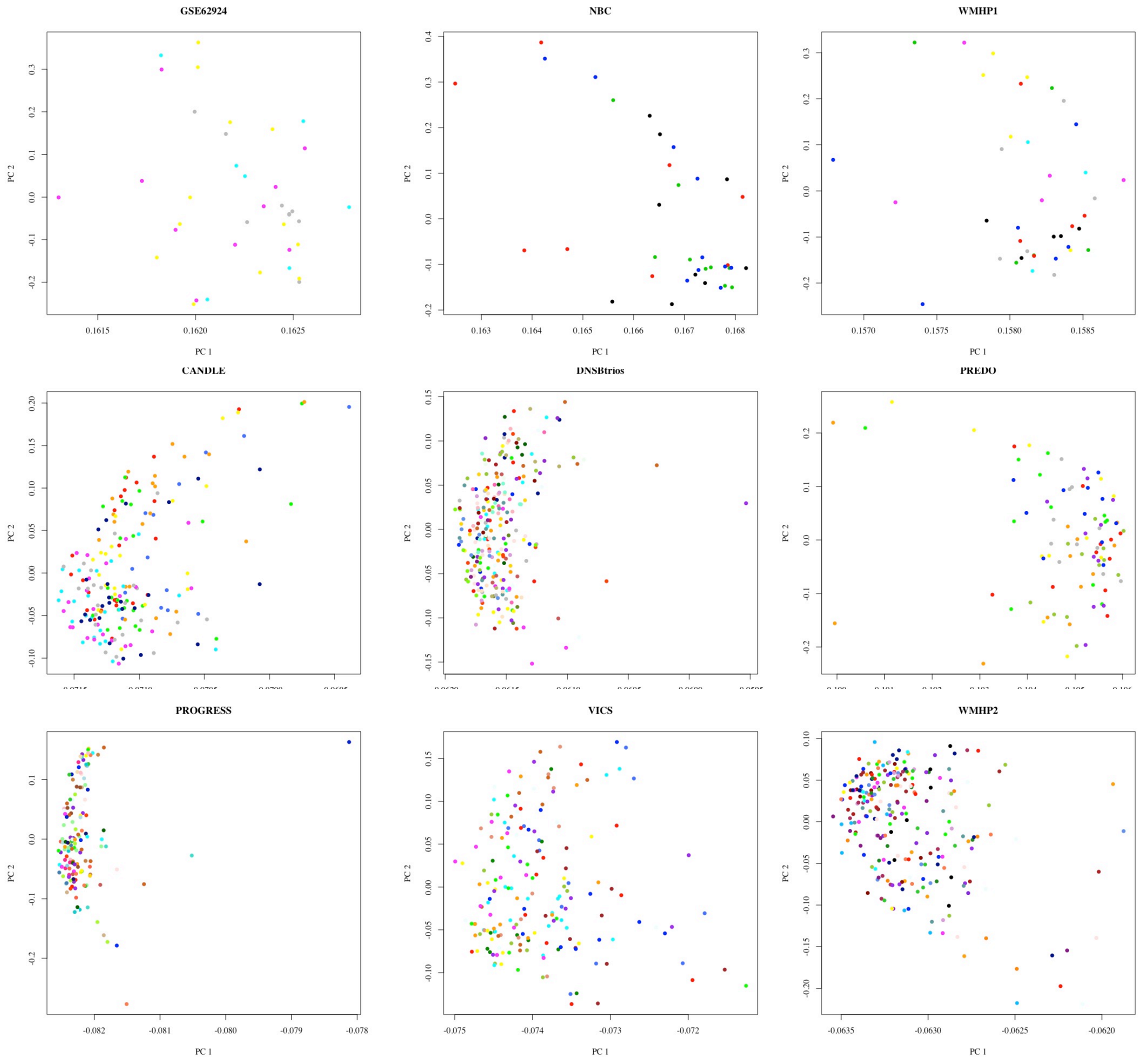

Cohort Descriptions

Emory Women's Mental Health Program (WMHP)

Women with a history of neuropsychiatric illnesses who participated in prospective studies to examine the perinatal course of illness, perinatal pharmacokinetic alterations, and impact of maternal stress on offspring were screened for inclusion in the current study. Details of this study has been described elsewhere [1-3]. Each woman's obstetrician estimated GA based on the date of her last menstrual period and ultrasound dating. Birth weight in kilograms was assessed at delivery and extracted from the medical records. Umbilical cord blood samples were collected at birth, stored on ice, and processed within 2 hours of delivery. Forty samples were run on the HumanMethylation450 array (abbreviated as WMHP1), and 251 were run on the HumanMethylation27 array (abbreviated as WMHP2). All women provided written informed consent prior to study enrollment following procedures approved by the Institutional Review Board of Emory University.

Conditions Affecting Neurocognitive Development & Learning in Early Childhood (CANDLE)

Neonates were selected from the Urban Child Institute's CANDLE study, a longitudinal cohort study of human development from pregnancy to age three conducted in Shelby County, Tennessee (Table 1). This cohort has been described in detail elsewhere [3-6]. A combination of obstetrician report (60%) or LMP (40%) was used to estimate GA. Whole umbilical cord blood samples were stored at 4^0C and processed

within 24 hours of delivery. Samples from this cohort were interrogated using the HumanMethylation27 BeadChip (N= 198).

Nashville Birth Cohort (NBC)

All subjects were recruited at Centennial Women's Hospital and the Perinatal Research Center in Nashville, TN beginning in 2003. Pregnant women were enrolled during their first clinical visit after obtaining informed consent as described previously [7]. Demographic and clinical data specific to the fetus was collected from clinical records. Gestational age of the neonate was determined by maternal reporting of the last menstrual period and corroboration by ultrasound dating. Umbilical cord blood samples were collected in EDTA tubes soon after placental delivery. Blood samples were centrifuged at 3,000 RPM to separate plasma, and buffy coats were aliquoted and stored at -80°C. Samples were processed on the HumanMethylation450 BeadChip (N=36).

Programming Research in Obesity, GRowth Environment and Social Stress (PROGRESS)

All participants were recruited at 12–24 weeks' gestation through the Mexican social security system after obtaining informed consent between 2007 and 2011. Women had to be greater than 18 years old that have an access to a telephone and a plan to reside within Mexico City for the following 3 years to be enrolled. The study was approved by the Institutional Review Boards of the participating institutions (Brigham and Women's Hospital and The National Institute of Public Health in Mexico). Gestational age was based on the difference between the birth date and the mother's report on enrollment of

her last menstrual period. Umbilical cord blood samples were aliquoted and frozen until manual DNA extraction including a red blood cell lysis step followed by isopropanol and ethanol extraction of DNA from total white blood cells. Resulting DNA samples were randomized for plating and bisulfite converted and analyzed on the HumanMethylation450 BeadChip by Illumina FastTrack Services (Illumina Inc., San Diego CA), prior to preprocessing and quality control with the methylumi package (N=148).

Victorian Infant Collaborative Study (VICS)

All 298 survivors born either <1000 g or <28 weeks' gestation in the state of Victoria were enrolled in a longitudinal follow-up study [8], which was approved by the Human Research Ethics Committees at the Royal Women's Hospital, the Mercy Hospital for Women, Monash Medical Centre, and the Royal Children's Hospital, Melbourne, Australia. Gestational age was determined by ultrasound estimation before 20 weeks of gestation, or by menstrual history in the minority if no ultrasound dating was available. DNA samples were derived from dried blood spots taken for newborn screening when infants were several days of age, after obtaining permission from the participants when they were aged 18 years, or from their parents if they were younger than 18 years. 183 samples were processed on the HumanMethylation450 BeadChip and passed sample quality control.

Danish neonatal screening biobank trios (DNSBtrios)

This cohort was recruited as a subset of samples in the Lundbeck Foundation funded initiative for integrative psychiatric research (iPSYCH). Trios (Mother/Father/child) were identified for a psychiatric study where all children and one or both parents had been diagnosed with a psychiatric illness (phenotype data not disclosed for the current study). All samples were isolated within the Danish Neonatal Screening Biobank (DNSB), which stores excess blood from the Danish Neonatal Screening Program. DNSB stores samples from almost every Dane born since 1982.

DNSBtrios study inclusion criteria was known GA as determined via last menstrual period until the late 1990s after which crown rump was used. The samples were also selected based on being collected relatively shortly after birth (<39 days).

The samples were collected via heel prick onto filter paper and then stored at -20°C. DNA was extracted from two punches of 3.2mm before being processed with the HumanMethylation450 BeadChip (N=264).

Prediction and Prevention of Preeclampsia (PREDO)

The mothers participating in the PREDO study come from one of ten hospital maternity clinics participating in the PREDO project in Finland [9]. They were recruited in conjunction of the first screening ultrasound at 12+0 to 13+6 weeks of gestation, based on which gestational age was also determined. Umbilical cord samples were collected in EDTA-tubes and stored immediately at -80°C. 91 samples were processed on the HumanMethylation450 BeadChip and passed sample quality control.

EpiPrem

Longitudinal samples were collected from neonates in the NICU at the Royal Women's Hospital in Melbourne, Australia. Blood collection occurred by heel stick and was collected on Whatman paper shortly after birth at 25 weeks gestation, one day post birth, and at the equivalent of 28, 32, 36, and 40 weeks' gestation (N=2).

Folic Acid supplementation in Pregnancy (FAP)

Healthy young (18-40 years old) pregnant women were recruited from Athens Regional Midwifery Clinic (Athens, GA) at their initial prenatal visit (<12 weeks gestation). In this study, the participants were selected based on the following exclusion criteria: 1) pre-existing chronic condition including anemia, diabetes or hypertension, 2) smokers, 3) those using prescription drugs, 4) those who were carrying more than one fetus. Participants were not allowed to take any vitamins/mineral supplements other than those provided by the research team for the study. Two doses of folic acid (400ug per day, 800ug per day) were provided to participants during gestation. The study regimen includes all the vitamins/minerals/DHA recommended for pregnant women. Cord blood samples were collected at delivery. 24 samples were processed on the HumanMethylation450 BeadChip and passed sample quality control.

1. Smith AK, Conneely KN, Newport DJ, Kilaru V, Schroeder JW, Pennell PB, Knight BT, Cubells JC, Stowe ZN, Brennan PA: **Prenatal antiepileptic exposure associates with neonatal DNA methylation differences.** *Epigenetics* 2012, **7:**458-463.
2. Schroeder JW, Smith AK, Brennan PA, Conneely KN, Kilaru V, Knight BT, Newport DJ, Cubells JF, Stowe ZN: **DNA methylation in neonates born to women receiving psychiatric care.** *Epigenetics* 2012, **7:**409-414.
3. Schroeder JW, Conneely KN, Cubells JC, Kilaru V, Newport DJ, Knight BT, Stowe ZN, Brennan PA, Krushkal J, Tylavsky FA, et al: **Neonatal DNA methylation patterns associate with gestational age.** *Epigenetics* 2011, **6:**1498-1504.
4. Adkins RM, Tylavsky FA, Krushkal J: **Newborn umbilical cord blood DNA methylation and gene expression levels exhibit limited association with birth weight.** *Chem Biodivers* 2012, **9:**888-899.
5. Mozhui K, Smith AK, Tylavsky FA: **Ancestry dependent DNA methylation and influence of maternal nutrition.** *PLoS One* 2015, **10:**e0118466.
6. Adkins RM, Krushkal J, Tylavsky FA, Thomas F: **Racial differences in gene-specific DNA methylation levels are present at birth.** *Birth Defects Res A Clin Mol Teratol* 2011, **91:**728-736.
7. Parets SE, Conneely KN, Kilaru V, Fortunato SJ, Syed TA, Saade G, Smith AK, Menon R: **Fetal DNA Methylation Associates with Early Spontaneous Preterm Birth and Gestational Age.** *PLoS One* 2013, **8:**e67489.
8. Roberts G, Cheong J, Opie G, Carse E, Davis N, Duff J, Lee KJ, Doyle L, Victorian Infant Collaborative Study G: **Growth of extremely preterm survivors from birth to 18 years of age compared with term controls.** *Pediatrics* 2013, **131:**e439-445.
9. Raikkonen K, Pesonen AK, O'Reilly JR, Tuovinen S, Lahti M, Kajantie E, Villa P, Laivuori H, Hamalainen E, Seckl JR, Reynolds RM: **Maternal depressive symptoms during pregnancy, placental expression of genes regulating glucocorticoid and serotonin function and infant regulatory behaviors.** *Psychol Med* 2015, **45:**3217-3226.
