# Supplementary material for: An epigenetic clock for gestational age at birth based on blood methylation data
Source: Genome Biol. 2016 Oct 7;17:206. doi: 10.1186/s13059-016-1068-z (PMC5054584; doi:10.1186/s13059-016-1068-z)
Supplement: Additional file 7: — Instructions for predicting gestational age from cord blood and blood spot methylation. (DOCX 137 kb) [file 13059_2016_1068_MOESM7_ESM.docx]

Instructions for predicting gestational age from cord blood and/or blood spot methylation

To predict gestational age (GA) from methylation data, we follow procedures suggested by Steve Horvath in Genome Biology 2013, 14:R115 doi:10.1186/gb-2013-14-10-r115.

Included with this file is R code that can be used to form the predictor; all code is adapted from the code provided by Horvath 2013. The main changes are that 1) a different set of CpG sites are used to form the GA predictor, and 2) we do not perform his transformation of age, since our predictor is designed to be used with neonates and infants only.

Files we provide in this supplement include:

**1) cgprobesGApredictor.csv (Additional File 3):** Our list of CpG probes and coefficients for calculating predicted GA.

**2) GAPredictor.R (Additional File4) :** A wrapper program that reads in user-supplied data (see below), calls **NormalizeAndPredictGA.R**, and writes predicted GA output to a file. May be modified by user to reflect filenames and pathnames of user-supplied data.

**3) NormalizeAndPredictGA.R (Additional File 5):** Normalizes data and performs GA prediction. This program will not normally require user modification.

Normalization: Uses a version of BMIQ (modified by Steve Horvath) to normalize methylation data prior to calculating predicted GA. Default normalization is set to TRUE in **GAPredictor.R**; normalization is highly recommended. Samples undergo a modified BMIQ normalization to a gold standard.

Prediction: Uses the information from **cgprobesGApredictor.csv** to fit the predictor.

**4) TestDataset.csv (Additional File 6):** A small demonstration dataset to provide an example of data format and allow testing and troubleshooting of the code. If the programs run successfully, the three samples should have DNAm GAs of 37.366, 38.346, and 39.324.

User must supply:

1) A dataset formatted similarly to TestDataset.csv – i.e., a data frame of beta values, saved in .csv format. The first column should contain CpG identifiers and should be labeled “CpGName”.

2) **Additional files 21, 22, and 24 from Horvath (2013)**, which may be obtained at <http://www.genomebiology.com/2013/14/10/R115/additional>

These files should not need any modification by the user. Additional file 23 is *NOT* needed, as a different set of CpGs is used to predict GA (provided in **cgprobesGApredictor.csv**, described above). Additional file 25 is also *NOT* needed, as we have replaced it with **NormalizeAndPredictGA.R** (which is similar, but does not perform the transformation needed for predicting adult age.)

To run programs:

1) Modify **GAPredictor.R** to provide filename and pathname for user-supplied methylation data (replace “TestDataset.csv” with correct filename).

2) Run **GAPredictor.R** (this program will call **NormalizeAndPredict.R**, which should be stored in the same directory.)

Output generated:

1) Log.txt records details about sample inputs and errors generated. If warning are generated, see log file for details.

2) Outputfile.csv is a dataframe of results. This contains the estimate of DNAm GA in weeks, and, if sufficient probes are supplied, predicted gender of sample.

3) dat0UsedNormalized.csv is a data frame of the normalized beta values.

Troubleshooting and common errors:

1) Note that if any of the 148 probes failed QC, and are not included in dat0, the predictor will be unable to predict DNAm GA.

2) Missing values should be coded as NA

3) length(tf) or length(newtf) returns 0 OR “CpG probes cannot be matched”

If length(tf) or length(newtf) returns 0, your dataset was not formatted correctly. **This is the most common problem.** Please ensure your data is in the format below, with CpG identifiers in the first column, not as row names. Once you have fixed this, reload probeAnnotation21kdatMethUsed, as it is written over in that block of code.

Format: Your data frame should have headers with column labels. The first column of your data frame for your input value should contain CpG names and should be labeled CpGName.

To check this once the data have been read in, you can enter “dat0[1:2,1:4]”

The output should appear similar to:

CpGName Sample1

1 cg01707559 0.28227360

2 cg02494853 0.01273777

3 cg03706273 0.02111593

4 cg04023335 0.15542060

4) “CpG probes cannot be matched” refers to probes missing from the gold standard file. These probes will be removed from your dataset.
